# Case report: Thromboembolic heartworm induced lower limb necrosis in a dog

**DOI:** 10.3389/fvets.2022.868115

**Published:** 2022-08-03

**Authors:** Piyanan Taweethavonsawat, Kasem Rattanapinyopituk, Kittipong Tachampa, Srireepong Kiertkrittikhoon, Wanarit Jitsamai, Wuthichai Klomkleaw, Nan Choisunirachon, Kiatpichet Komin

**Affiliations:** ^1^Parasitology Unit, Department of Pathology, Faculty of Veterinary Science, Chulalongkorn University, Bangkok, Thailand; ^2^Biomarkers in Animal Parasitology Research Group, Chulalongkorn University, Bangkok, Thailand; ^3^Pathology Unit, Department of Pathology, Faculty of Veterinary Science, Chulalongkorn University, Bangkok, Thailand; ^4^Department of Physiology, Faculty of Veterinary Science, Chulalongkorn University, Bangkok, Thailand; ^5^Department of Surgery, Faculty of Veterinary Science, Chulalongkorn University, Bangkok, Thailand; ^6^Department of Anatomy, Faculty of Veterinary Science, Chulalongkorn University, Bangkok, Thailand

**Keywords:** aberrant, dog, heartworm, ischemia, parasitic thrombosis

## Abstract

A 9-year-old spayed female boxer suffered from lameness in both hindlimbs with a perforated paw wound. Additionally, a linear, worm-like creature was penetrating out from the wound. On examination, the dog was emaciated and infected with heartworms, detected through a fresh blood smear, echocardiography, and transabdominal ultrasonography. Adult heartworms were detected at the right atrium (RA), right ventricle (RV), and pulmonary artery (PA), including the distal abdominal aorta, external iliac, and femoral arteries. During the surgery, adults heartworms were removed from both the heart (*n* = 8) and the femoral arteries (*n* = 5). Unfortunately, not all heartworms could be removed from these locations due to the extent of the heartworm infection. The opened, ischemic wounds in the distal limbs progressively expanded and the dog subsequently died, possibly due to caval syndrome complications and septicemia. The necropsy showed no evidence of an atrial septal defect, and a total of 25 adult heartworms were collected from the perforated paw, heart, pulmonary, and femoral arteries. All worms collected during the necropsy process were molecularly confirmed to be *Dirofilaria immitis*.

## Introduction

Canine heartworm disease, caused by *Dirofilaria immitis*, is distributed worldwide but is more common in tropical areas. Recently, using microscopy, the prevalence of *D. immitis* was reported to be 0.43% in Bangkok, Thailand (1). Many species of culicine mosquitoes, such as *Culex* spp., *Aedes* spp., and *Anopheles* spp., act as vectors for the completion of the heartworm life cycle. When taking a blood meal from a microfilaremic host, the mosquitoes become infected and the microfilariae develop into the third-stage larvae (L3) in the Malpighian tubules of the mosquitoes. Subsequently, the L3 migrates to the host while the mosquito is suckling blood and completes the cycle by developing into an adult heartworm within 7–9 months in the pulmonary arteries (PAs), right atrium (RA), and right ventricle (RV) (2).

The adult heartworm has been reported at ectopic sites, such as the brain, abdominal cavity, and abdominal aorta, including its branches (Supplementary Table 1) (3–6); however, only one report (7) has described aberrant adult heartworm induced severe ischemia by the occlusion of extremity vasculature. The current report provides additional evidence of ectopic heartworm disease in a dog that was infected with many adult heartworms, subsequently causing aberrant migration and inducing severe limb ischemia. This article provides information on clinical signs, examination procedures, minimally invasive techniques for removal of adult heartworm from the heart and ectopic sites, and heartworm confirmation techniques, such as necropsy and microscopic lesions, for dogs with aggressive *D. immitis* infections.

## Case presentation

A 9-year-old, 25 kg, the spayed female boxer was presented due to a complaint of paresis and lameness of both hindlimbs for a week. Initial physical examination revealed that the dog was severely emaciated with a body condition score of 1/9. The hydration status and lung sound were normal, however, a grade 4/6 diastolic murmur at the right base of the heart was detected as well as a weak femoral pulse. Additionally, appetite was normal with no evidence of exercise intolerance or coughing. Orthopedic and radiographic examinations of the hindlimbs were performed with unremarkable results. The dog was then treated with meloxicam (0.1 mg/kg, PO, SID, Metacam^®^).

The clinical signs, especially the lameness of both hindlimbs, persisted until the follow-up period a week later, with progressive bruise-necrosis wounds, particularly at the footpad of the right hindlimb (Supplementary Figure 1). A white to yellowish linear, worm-like organism extruded from the necrotic open wound. Further history revealed that the dog was raised in an orchard. Despite a history of rabies vaccination, the dog had never received microfilarial prophylaxis.

**Figure 1:**
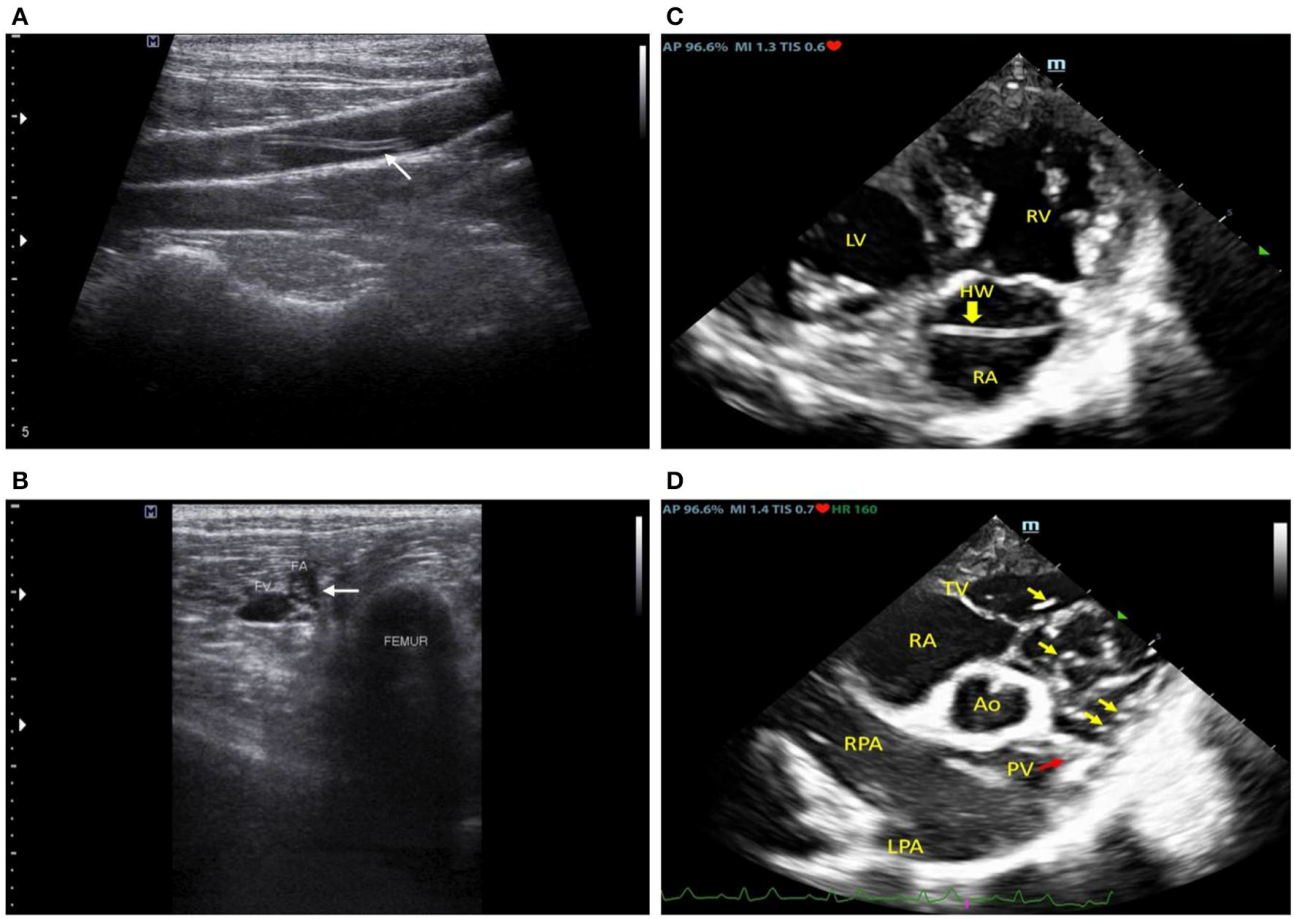
Ultrasonographic images at the left external iliac artery **(A)**, mid femoral artery **(B)**, and echocardiographic images **(C,D)** revealed linear hyperechoic structures in the cardiovascular lumen which were subsequently molecularly confirmed to be *Dirofilaria immitis*.

Upon the second visit, general health status was similar to the first presentation; however, hematology profiles indicated leukocytosis (neutrophilia) and a positive result for microfilaria (Supplementary Table 2). Therefore, the dog was treated with antimicrobial doxycycline (10 mg/kg, bid, PO, Vibramyclin^®^) and selamectin spot-on (Revolution^®^). The dog was then subjected to further imaging examinations of intrathoracic and intra-abdominal organs by radiography and ultrasonography. Both the right lateral and ventrodorsal thoracic radiographs were performed using a digital radiograph X-ray machine (Brivo DR-F, GE Healthcare, Beijing, China); the results indicated right heart enlargement with prominent pulmonary knob and mild enlargement of the PA. Transabdominal ultrasound was performed using a 12 MHz-linear transducer (Accuvix XG, Medison, Seoul, Korea). This revealed a coarse hyperechoic hepatic parenchyma and a focal, ill-defined, hypoechoic nodule (1.41 cm) at the splenic tail. Furthermore, hyperechoic, linear worm-like structures were found at the bifurcation of the abdominal aorta, the external iliac arteries (Figure 1A), the distal femoral arteries near the distal femur (Figure 1B), and the saphenous arteries of both hindlimbs.

Following that, an echocardiography examination was performed using a 2–4 MHz phased array probe (M9, Mindray^®^, Shenzhen, China). The 2D echocardiography images (Figure 1C) revealed an enlarged RA and RV with a flattened interventricular septum (IVS), suggesting eccentric hypertrophy and volume overload of the RV. Multiple hyperechogenic lines were observed in the RA, RV, and PA, indicating the presence of adult heartworms (Figure 1D). The main PA (MPA) and the right PA were severely enlarged with an MPA/Ao ratio of 1.2 on the short axis view. Pulmonic valve leaflets were of irregular shape and severely thickened, with moderate pulmonary regurgitation (PR). Systolic pulmonary arterial pressure gradient was 20 mmHg and PR maximal pressure gradient was 25 mmHg. Heartworms were seen to repeatedly translocate between the RV and PA during the cardiac cycle as recorded in the cine clip (Supplementary Video 1). The left atrium (LA) was mildly enlarged (LA/Ao = 1.7) and the tricuspid valves appeared to be mildly thickened with no observable amount of tricuspid regurgitation. Pulmonary hypertension (PH) was diagnosed with the right heart remodeled due to many heartworm infections. Additionally, neither an atrial septal defect (ASD) nor perforated foramen ovale (PFO) of the heart was detected based on echocardiography.

Since many adult heartworms were detected at the heart and both hindlimbs, which later caused distal limb necrosis, it was planned for the dog to undergo surgical removal of worms from both sites. A week prior to surgery, meloxicam was stopped to prevent the anti-coagulation effect, and the blood profile was rechecked. Before surgery, food and drink were withheld from the dog for 8 h. The dog was premedicated with 0.2 mg/kg of midazolam (Midazolma, Hexal^®^) and was subsequently administered 10 mg/kg of fentanyl (Fentanyl-Hameln^®^). Following endotracheal tubulation, the anesthetic condition of the dog was maintained with 2–5% of isoflurane. Additionally, the dog was injected with dexamethasone (4 mg/kg; IV, Lodexa^®^) and chlorpheniramine (0.5 mg/kg; IM, ANB®) to prevent transfusion reaction during the surgical intervention, which was given during the operation.

**Figure 2:**
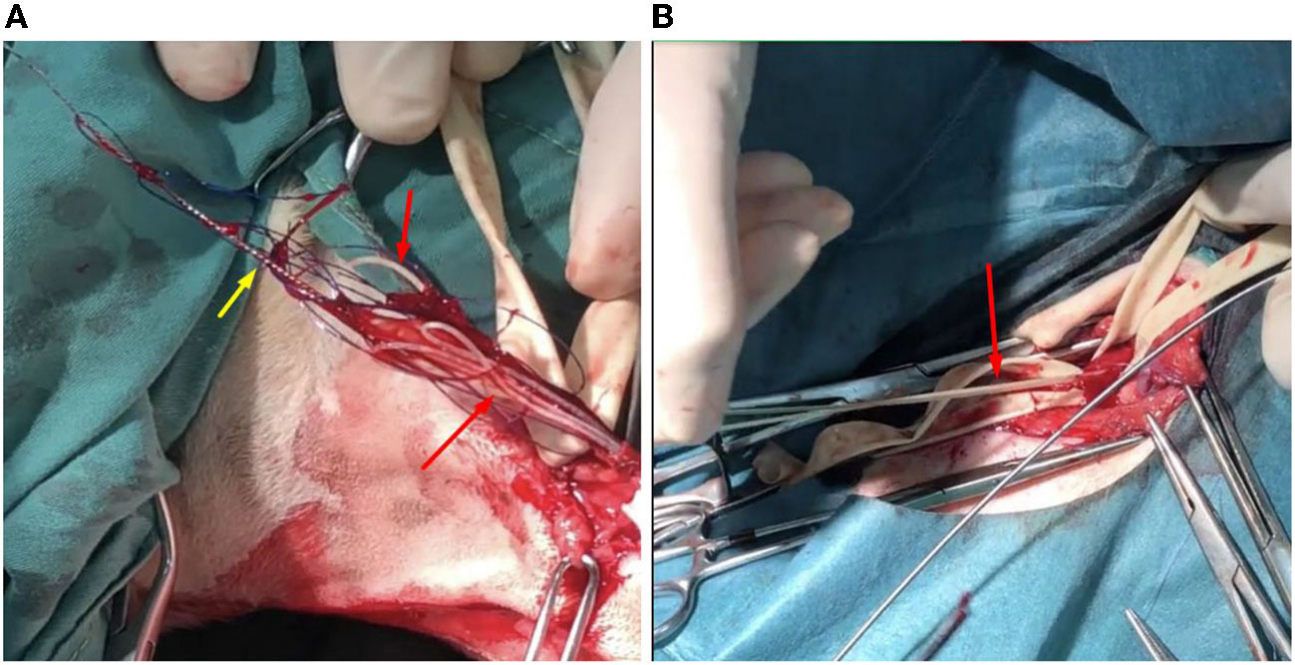
The photographic images displayed the adult *Dirofilaria immitis* (red arrows) removal from the jugular vein **(A)** and the femoral artery **(B)**. At the jugular vein, a modified, brush-like instrument (yellow arrow) was used to take off the cardiac heartworms.

The surgical procedures began with cardiac heartworm removal *via* left jugular vein phlebotomy. After the skin incision, 2 Penrose drains (diameter: 1/4 inch) were looped around the jugular vein to prevent bleeding after the phlebotomy. Cardiac heartworm extraction from the RV was performed under fluoroscopy (BV Pulsera, Philips Health, Best, Netherland) using a blush-like instrument made from small diameter Kirshner wire (no. 26) with monofilament, non-absorbable polyamide suture material (size 3/0, Dafilon, B. Braun, Ruby, Spain) (Supplementary Figure 2). The instrument was inserted into the jugular vein and pushed into the right ventricle. After twisting the instrument several times and then withdrawing it, the adult heartworms were found attached to the phlebotomy line (Figure 2A). The procedure was repeated several times before the closure of phlebotomy by standard procedure using monofilament absorbable glyconate suture material (size 3/0, Monosyn, B. Braun, Ruby, Spain). Eight cardiac heartworms were collected during this procedure.

Subsequently, extraction of lower limb heartworms from the left and right femoral arteries was performed. After aseptic technique preparation, skin incisions above the left and right groins were performed. Femoral arteries at these locations were obviously thickened. Penrose drains (diameter: ¼ inch) were looped around the femoral arteries prior to the arteriotomy. Femoral arteries were cut linearly by a surgical blade for a 1 cm incision and alligator forceps (Microsurgical Grasping Forceps, 1.4 mm of tip width, and 6 inches of shaft length) were used to remove adult heartworms from femoral and iliac arteries (Figure 2B). The procedure was repeated several times, and closures of arteriotomy and skin incision lines were performed with a standard procedure similar to that used in the cervical area. From both limbs, five heartworms were collected. Post-operatively, the dog was treated with marbofloxacin (5 mg/kg; IV, Marbocyl^®^) and tramadol (5 mg/kg; SC, Pharmadol^®^).

**Figure 3:**
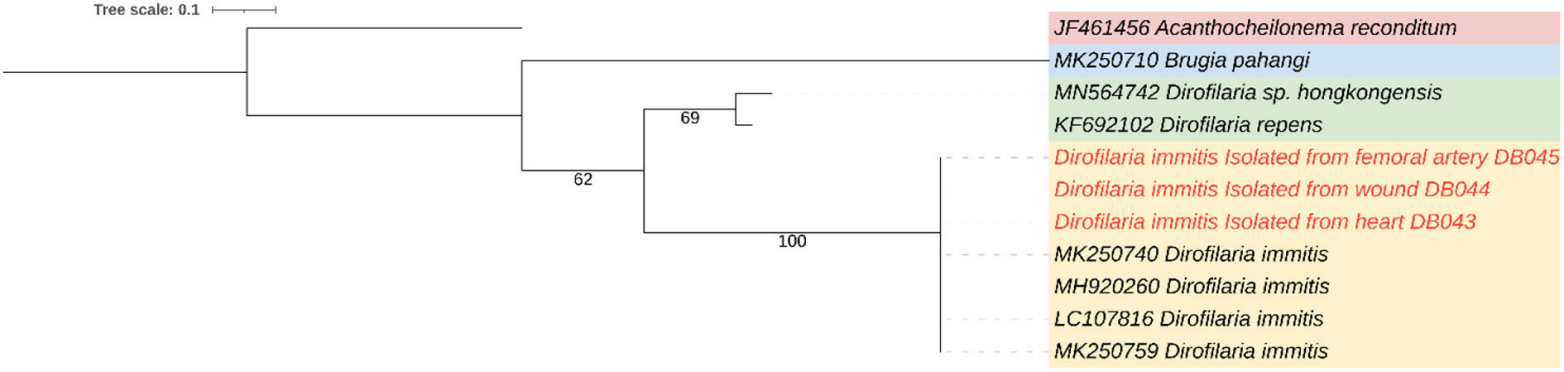
A maximum-likelihood phylogenetic tree showing the genetic relation between *Dirofilaria immitis* from heart, femoral artery, and wound (red color) and other filarial species using cytochrome oxidase subunit I gene.

The worms were slender and white from both cardiac and hind limbs. They were subjected to further laboratory analysis. This determined that only female adults were obtained from the heart and femoral arteries. Nematodes were sized at an average of 14.97 ± 1.34 cm. To confirm the species of adult nematodes from each location, including the worm previously taken from the tarsal pad wound, all worms were preserved in 70% ethanol and subjected to DNA extraction according to the manufacturer's (Qiagen^®^) protocol. PCR was conducted targeting cytochrome oxidase subunit I regions using primers COI-int-F (5′TGATTGGTGGTTTTGGTAA′3) and COI-int-R (5′ATAAGTACGAGTATCAATATC′3) (8). The PCR reaction mix comprised 10 μl of 2x ViRed Taq Master Mix (Vivantis, Malaysia), 0.5 μM of each primer, and 2 μl of DNA. The conditions were as follows: 40 cycles at 94°C for 45 s, 52°C for 45 s, 72°C for 90 s, and 94°C for 3 min for pre-denaturation, with a final extension step at 72°C for 7 min (8). The PCR products were subjected to gel electrophoresis, purified, and sequenced. The genetic identification revealed that all nematodes were *D. immitis* (Figure 3).

After the surgical removal of adult heartworms, the dog received post-operative care; surgical wounds and necrotic foot pad wounds were dressed daily and antibiotic and analgesic treatments were administered. However, 6 days postoperatively, leukocytosis with hypoglycemia and progressive infarction, and infection of both distal limbs were detected (Supplementary Table 2). Therefore, hemoculture was performed with a conventional technique by aseptically collecting 3 ml blood from a cephalic vein, with double samples, showing that the dog was infected with *Enterobacter cloacae*, which was sensitive to imipenem. Therefore, imipenem-cilastatin (5 mg/kg, IV, q 8h, Tienam^®^) was administered, however, the vital and laboratory signs, especially hematological results, of the dog, did not improve. In addition to antibiotic treatment, the dog continued treatment with tramadol and was further given parenteral fluid (Dextrose 5% in Water, Thai Otsuka^®^) with additional glucose (Glucose 50%; ANB^®^) for the correction of hypoglycemia, such as packed red blood cell transfusion (220 ml) for anemia, and oral supplements with albumin tab (PowerPlus^®^), silymarin (20 mg/kg, Pharmarin^®^), ursodeoxycholic acid (20 mg/kg, Ursolin^®^), and iron (III) hydroxide polymaltose complex with folic acid (Eurofur^®^). The dog died 9 days after surgical treatment, and a necropsy was performed to investigate further.

**Figure 4:**
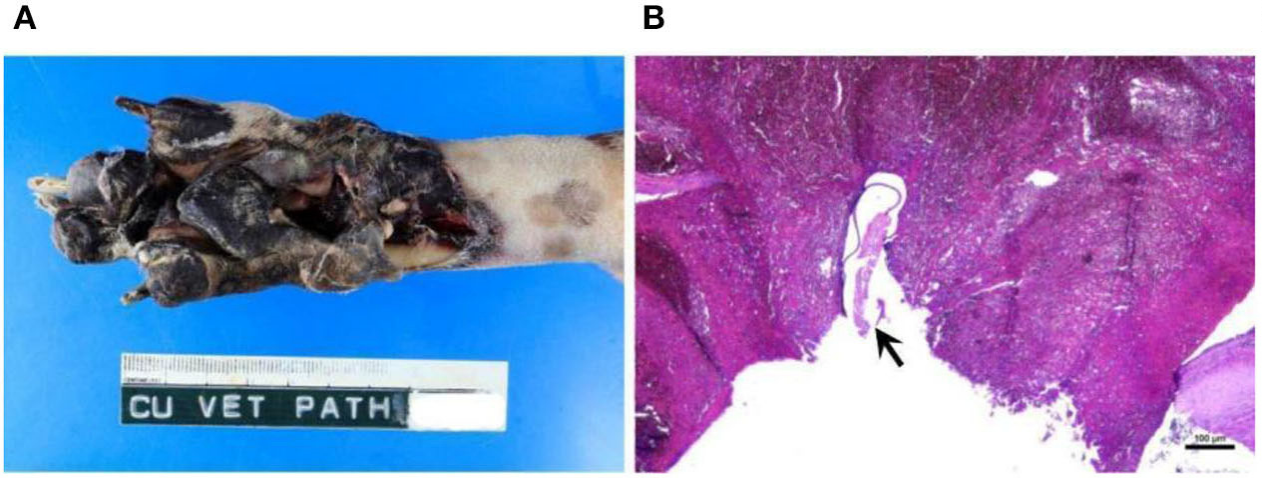
Pedal gangrenous necrosis was demonstrated in the metatarsal-phalangeal region of the hindlimb **(A)**. Microscopically, occluding thrombus is demonstrated in the artery with the presence of a cross-section of the worm, ~100–250 μm in size (arrow) **(B)**. Bar = 100 μm.

Before the necropsy, the carcass was scored to have moderate autolysis. A significant gross lesion observed during the external examination was restricted to the paws of both hindlimbs and pedal gangrenous necrosis was demonstrated in the metatarsal-phalangeal regions (Figure 4A). Heartworms were taken from the gangrenous wounds of both paws. Necropsy findings of the internal organs showed moderate cardiomegaly of the right heart with occupying worms in the RA and RV, extended to the PA. Thickening and irregularity of the pulmonic valve and pulmonary trunk were observed (Supplementary Figure 3). In line with the echocardiography result, ASD was not detected. An occluded thrombus was seen to be lodged in the femoral artery near the hip joint. Foci of infarcts were observed at the head of the spleen (Supplementary Figure 4) and the right kidney. Hepatomegaly was seen with yellowish discoloration, and the cut hepatic surface demonstrated a nutmeg liver pattern. During the necropsy, 25 adult heartworms were collected from the heart, pulmonary arteries, and hind limb vasculatures.

Microscopically, multiple thrombi were found in various organs, such as pulmonary and splenic vessels, as well as the femoral artery. Heartworms were also observed inside several obstructive thrombi (Figure 4B). The cross-section of the worm was ~75 μm ×200 μm. The PA and pulmonary trunk were found to have severe fibrinosuppurative endoarteritis. Other microscopic findings included moderate hypertrophic cardiomyopathy of both ventricles, with mild focal suppurative myocarditis and focal myocardial necrosis, moderate chronic multifocal fibrosing pneumonia, moderate hepatic lipidosis, dilated hepatic sinusoid with an accumulation of erythrocytes and leukocytes, severe diffuse proliferative glomerulonephritis (Supplementary Figure 5) with multifocal lymphoplasmacytic interstitial nephritis, and massive splenic necrotic tissue consistent with infarction.

## Discussion

Most ectopic adult heartworm infections are reported on the left side of the circulatory system, including the distal abdominal aorta or extending to the femoral artery (3, 5, 6, 9, 10). However, only one report (7) has previously demonstrated occlusion of the extremity vasculatures by aberrant adult heartworm. The current report is further evidence of ectopic heartworms in a dog infected with many adult heartworms, which subsequently caused abnormal migration and severe limb ischemia. To the best of our knowledge, the migratory pathway to arterial systems in dogs remains unclear. However, some parasites can cause cardiopulmonary diseases in felids, such as *Capillaria aerophila, Triglostrongyus brevior*, and *Angiostrongylus chabaudi*, but these have only been reported in European wildcats (11). The proposed hypothesis of ectopic heartworm infection includes the aberrant migration of L5 worms and the relocation of adult worms *via* cardiovascular shunting (12). Although the right to left shunting was demonstrated in some ectopic heartworm cases (3, 6), no report has demonstrated direct evidence of the atrial septal defect. Additionally, the prevalence of patent foramen ovale with the right to left shunting and pulmonic stenosis was reported in dogs ranging from 4 to 93 months old (13), while young adult heartworm was reported in the pulmonary artery within 6 month-old dogs (14). The migration might have occurred before the closing of the foramen ovale.

Echocardiography is a useful diagnostic tool for detecting adult heartworms in the PA and in the heart chambers. Additionally, echocardiographic signs of pulmonary arterial hypertension due to heartworm infection can be shown using this technique (15). In this case, we observed many heartworms that were localized and translocated in the PA, RV, and RA (Supplementary Video 1). This was consistent with the number of heartworms collected during surgical extraction and necropsy. According to recent American College of Veterinary Internal Medicine (ACVIM) consensus guidelines (15), several echocardiographic signs of PH were observed (i.e., enlarged PA, flattening of the IVS, RV hypertrophy, RA enlargement, and systolic notching of the Doppler RV outflow) which indicated a high probability of PH in this dog. In the current report, echocardiography did not identify the presence of an ASD and/or PFO in this dog. Therefore, the mechanism by which heartworms migrated to the left side of the circulatory system remains unclear in this case. The severe thickening of pulmonic valves may have resulted from the irritation caused by the worms and the consequent inflammatory processes. This led to an increase in resistance and workload of the RV that caused the remodeling.

We speculate that the open wound at the tarsal pad resulted from the penetration of the adult worm through the distal limb vasculature. The obstructing worm caused poor perfusion of the cranial tibial artery, resulting in arteriolitis and a necrotizing wound. Cats have also been presented with ectopic heartworm as well as dogs (12). The clinical signs of ectopic heartworm disease depend on where the occlusion has occurred. Most cases reported the occlusion in the distal abdominal aorta and femoral artery, which resulted in lameness and paralysis of the hindlimb (3, 5, 6).

In addition to the clinical signs, ultrasonography is a common method for the detection of aberrant adult heartworms, especially in the distal abdominal aorta, external iliac, and femoral arteries (6, 9, 10, 12). Ultrasonography easily detects heartworms in larger vessels that are seated in a deep location. Compared with the distal abdominal aorta and external iliac artery, worm detection at the smaller vasculature, such as the femoral artery and its branches, are more challenging. These vessels are at superficial locations and the size of the worm may fit the vessel diameter; a medium-MHz transducer used for abdominal ultrasonography may not be suitable for detection. In addition to ultrasonography, angiography and contrast enhanced-computed tomography have been used to assist in visualizing the embolism and the ischemic region (6, 12).

According to the hematological profile, leukocytosis with neutrophilia and anemia were present. Leukocytosis was relevant to the hindlimb infarction and inflammation due to the arterial thrombosis and it suggested the occurrence of septicemia. Clinical chemistry results showed an increase in ALT and ALP. The damage to hepatocytes due to anemia may be responsible for the increase in ALT. Increased ALP may have resulted from intrahepatic cholestasis due to the fatty degeneration of hepatocytes (hepatic lipidosis) that was microscopically observed. Furthermore, microfilaria was not detected in the blood profile following the surgical removal of heartworms. This might have been caused by several etiologies, such as the preoperative treatment with topical selamectin or the sensitivity of the detection method. In this case, a fresh blood smear was used to detect the blood parasite. Therefore, to conclude that a dog is absent of microfilaria, a Knott's test should be performed.

To remove adult heartworm from the canine or feline RV, several instruments have been used, such as a basket (16), tripod grasping forceps (16), homemade snare (17), string-type horsehair brush (18), or alligator forceps (18), however, these instruments were not available in this case. Therefore, a novel, homemade, brush-like instrument comprised of small-sized Kirschner wire and suture was an effective instrument to remove the worms and the instrument was easily detected under fluoroscopy. However, the matching of instrument and patient sizes should be further evaluated for its proper use. In addition to cardiac heartworm removal, the femoral artery was revealed to be stiff and hard. This might have resulted from irritation by the worms and induced arteriolitis (10).

Despite the operations being successfully performed, complete removal of the worms could not be achieved for two reasons. First, the worms were localized in an area (i.e., pulmonary artery and cranial tibial artery), which our instrument could not reach. Second, chronic vasculitis of the femoral artery caused a reduction in its diameter resulting in the instrument only being able to insert a short distance from the incision. Therefore, the ischemia at the lower area of both hindlimbs progressed and heightened the risk of infection. In this case, *Enterobacter cloacae*, a Gram-negative, facultatively-anaerobic, bacilli bacterium, was detected through hemoculture. This bacterium is normally found as gut flora and can be considered a nosocomial infection, especially in immunocompromised patients, such as patients with cancer (19) and neonates (20). The positive culture result could have been due to contamination as only one blood culture sample was obtained. It has been reported that infections from this bacterium have occurred through contaminated medical equipment (21, 22). This dog suffered from chronic heart disease resulting in poor body condition scores and low serum protein, which indicate that the dog was unhealthy and immunocompromised (23). Additionally, no cancer was detected in this patient. The progressive ischemia open wound at the lower limb was suspected to be the origin of the infection, however, one limitation of this case report is that bacterial culture from the ischemic open wound was not performed.

Caval syndrome (CS) is a specific condition in which a mass of heartworms localized in the RV, RA, or vena cava results in tricuspid insufficiency and compromised RV filling. Common clinical findings in dogs with CS include weak peripheral pulse, loud tricuspid murmur, icterus, pale mucous membrane, and hemoglobinuria. Although the dog presented in this report had most clinical indicators of CS, hemoglobinemia and hemoglobinuria were not observed. We speculate the cause of death in this dog may possibly be due to (1) cardiovascular failure because of CS based on clinical findings and autopsy or from and (2) septicemia based on the hemoculture result.

## Conclusion

Heavy heartworm infection can cause aberrant migration of worms into ectopic locations. Although the mechanism of migration cannot be explained in this case, this report showed that a large amount of aberrant heartworm migration, especially to the distal limb, can cause worm embolisms and, subsequently, impede blood circulation, causing fatal necrosis. Removing worms from the distal limb is difficult due to the small size of the anatomical structures. Therefore, early detection and treatment are ideal. Prevention and control require the pet owner to be advised of these conditions to avoid cases of ectopic heartworms.

## Data availability statement

The original contributions presented in the study are included in the article/Supplementary material, further inquiries can be directed to the corresponding author/s.

## Ethics statement

Ethical review and approval was not required for the animal study because author informed consent was obtained from the owner for the participation of their dog in this study. Written informed consent was obtained from the owners for the participation of their animals in this study.

## Author contributions

PT, KR, KT, NC, and KK performed the concept/design, data analysis/interpretation, drafting article, and critical revision of the article. SK and WK performed a critical revision of the article and approved the article. WJ performed the laboratory analysis and approved the article. All authors contributed to the article and approved the submitted version.

## Funding

This work was supported by the Special Task Force for Activating Research, Chulalongkorn University (STF 6401531001-1). The clinical data of this report was supported by the Small Animal Hospital, Faculty of Veterinary Science, Chulalongkorn University.

## Conflict of interest

The authors declare that the research was conducted in the absence of any commercial or financial relationships that could be construed as a potential conflict of interest.

## Publisher's note

All claims expressed in this article are solely those of the authors and do not necessarily represent those of their affiliated organizations, or those of the publisher, the editors and the reviewers. Any product that may be evaluated in this article, or claim that may be made by its manufacturer, is not guaranteed or endorsed by the publisher.

## Supplementary material

Supplementary Figure 1 Photographic image of the right caudal palmar showed the necrotic small wounds above the tarsal pad.

Supplementary Figure 2 A blush-like instrument for intracardiac worm removal, made from small diameter Kirshner wire (no. 26) with monofilament, non absorbable polyamide suture material.

Supplementary Figure 3 The thickening and irregularity of the pulmonic valve and pulmonary trunk were demonstrated (arrow).

Supplementary Figure 4 Focal infarction (area in the dashed line) was grossly observed at the head of the spleen **(A)**. Microscopically, the occurrence of the cross-sectional adult heartworm parasite in the splenic vessel (arrow). The heartworm was characterized by ~75 μm ×200 in size with 5-μm-thick internal ridges of the cuticle, a well-developed muscle layer, and small guts **(B)**. Bar = 50 μm.

Supplementary Figure 5 Proliferative glomerulonephritis was diffusely observed in the renal cortex of both kidneys. Glomeruli were hyperplasia characterized by increased cellularity and enlarged (arrows). Bar = 25 μm.

Supplementary Video 1 A right parasternal short axis view at the pulmonary trunk level showed translocation of heartworms (as multiple hyperechoic lines) repeatedly between the right ventricle and pulmonary artery during the cardiac cycle as recorded in the cine clip.
