# Supplementary material for: Case report: Thromboembolic heartworm induced lower limb necrosis in a dog
Source: Front Vet Sci. 2022 Aug 3;9:868115. doi: 10.3389/fvets.2022.868115 (PMC9382088; doi:10.3389/fvets.2022.868115)
Supplement: Supplementary file 1 [file Table_1.docx]

Table 1. Cases presenting ectopic heartworm disease in dogs (supplement).

| **Signalments** | **Clinical signs** | **Location of adult heartworm** | **Reference** |
| --- | --- | --- | --- |
| 5-year-old Terrier | Progressive lameness, swelling, and pain in the left hindlimb and paralyzed in the right hindlimb. | Femoral arteries, right side of the heart and pulmonary arteries. | (Slonka et al., 1977) |
| 3 dogs were reported | Unstable gait, circling, tumble, roaring, visual disorders, disturbed swallowing, cloudiness of consciousness and coma | Cerebral artery | (Patton and Garner, 1970) |
| 3-year-old, male Siberian Husky | Sudden right hind-leg lameness | Right femoral artery | (Burt et al., 1977) |
| 6-year-old, intact male German Shepherd | Non-weight-bearing lameness of the right hind- leg and paresthesia of both hind paws manifested as excessive licking and chewing of the digits. A cough of several months | Abdominal aorta extending from the diaphragm, left femoral artery and right femoral artery. | (Frank et al., 1997) |
| 2-year-old,  castrated male Labrador Retriever | Right hindlimb lameness and exercise intolerance. | Distal abdominal aorta and femoral arteries | (Frank et al., 1997) |
| 9-year-old,  intact male mixed breed | Acute onset of hindlimb pain and weakness | Abdominal aorta extending from the renal arteries to the left and right external iliac arteries | (Frank et al., 1997) |
| 4-year-old,  intact male  wolf hybrid | Left hindlimb lameness and pedal paresthesia | Internal iliac and femoral arteries | (Frank et al., 1997) |
| 6-year-old,  intact male Cocker Spaniel | History of coughing, lethargy, exercise intolerance, and paresthesia of the right hind paw. non-weight- bearing lameness of the right hindlimb and had necrotic lesions on the right hind paw. | Distal aorta and right femoral artery | (Frank et al., 1997) |
| 2-year-old, castrated male Dachshund | Vomiting and hematochezia | Abdominal aorta from just cranial to the right renal artery to the iliac bifurcation | (Grimes et al., 2016) |
| 3-year-old, female mongrel | No clinical signs | Abdominal cavity | (Oh et al., 2008) |
| 5-year-old, intact male mixed-breed | N/A | Abdominal aorta and inflammatory hepatic nodules | (Goggin et al., 1997) |
