# Supplementary material for: Case report: Thromboembolic heartworm induced lower limb necrosis in a dog
Source: Front Vet Sci. 2022 Aug 3;9:868115. doi: 10.3389/fvets.2022.868115 (PMC9382088; doi:10.3389/fvets.2022.868115)
Supplement: Supplementary file 2 [file Table_2.DOCX]

Table. 2 Hematology and serum biochemistry profiles of a heartworm infestation dog at before operation and 6 days post-operation.

| **Parameters** | **Units** | **Normal range** | **7 days Before operation** | **6 days**  **Post-operation** |
| --- | --- | --- | --- | --- |
| Red blood cell | x 106 per ul | 5.20 - 8.06 | **2.99** | **3.77** |
| Hemoglobin | g/dl | 12.4 - 19.1 | **7.1** | **8.8** |
| Hematocrit | % | 29.8 - 57.5 | **20.3** | **25.9** |
| MCV |  | 64.7 -72.0 | 67.9 | 68.5 |
| MCH |  | 22.2 - 25.4 | 23.6 | 23.4 |
| MCHC |  | 34.0 - 36.6 | 34.8 | 34.2 |
| White blood cell | per ul | 5.4 - 15.3 | **22.03** | **27.92** |
| Platelet | x 103 per ul | 160 - 525 | 516 | 30 |
| Neutrophils | per ul | 3000 - 11500 | **20509** | **25686** |
| Bands | per ul | 0 - 300 | 0 | 0 |
| Eosinophils | per ul | 100 - 1250 | **22** | **28** |
| Basophils | per ul | rare | 0 | 28 |
| Lymphocytes | per ul | 1000 - 4800 | 1167 | 1061 |
| Monocytes | per ul | 150 - 1350 | 396 | 1116 |
| Blood parasite | - | - | Microfilaria | Not found |
| ALT | units | 4 - 91 | **4155** | **204** |
| ALP | units | 3 - 60 | **1170** | **2083** |
| BUN | mg% | 7 - 26 | 18.7 | **33.2** |
| Creatinine | mg% | 0.6 - 1.4 | 0.8 | 1.2 |
| Total protein | g% | 5.8 - 7.9 | 6.8 | **5.3** |
| Albumin | g% | 2.6 - 4.0 | **2.1** | **1.1** |
| Glucose | mg/dL | 60- 110 | **-** | **42** |

MCV: mean cell volume; MCH: mean corpuscular hemoglobin; MCHC: mean corpuscular hemoglobin concentration; ATL: alanine aminotransferase; ALP: alkaline phosphatase; BUN: blood urea nitrogen
