# Supplementary figures and images for: Case report: Thromboembolic heartworm induced lower limb necrosis in a dog
Source: Front Vet Sci. 2022 Aug 3;9:868115. doi: 10.3389/fvets.2022.868115 (PMC9382088; doi:10.3389/fvets.2022.868115)

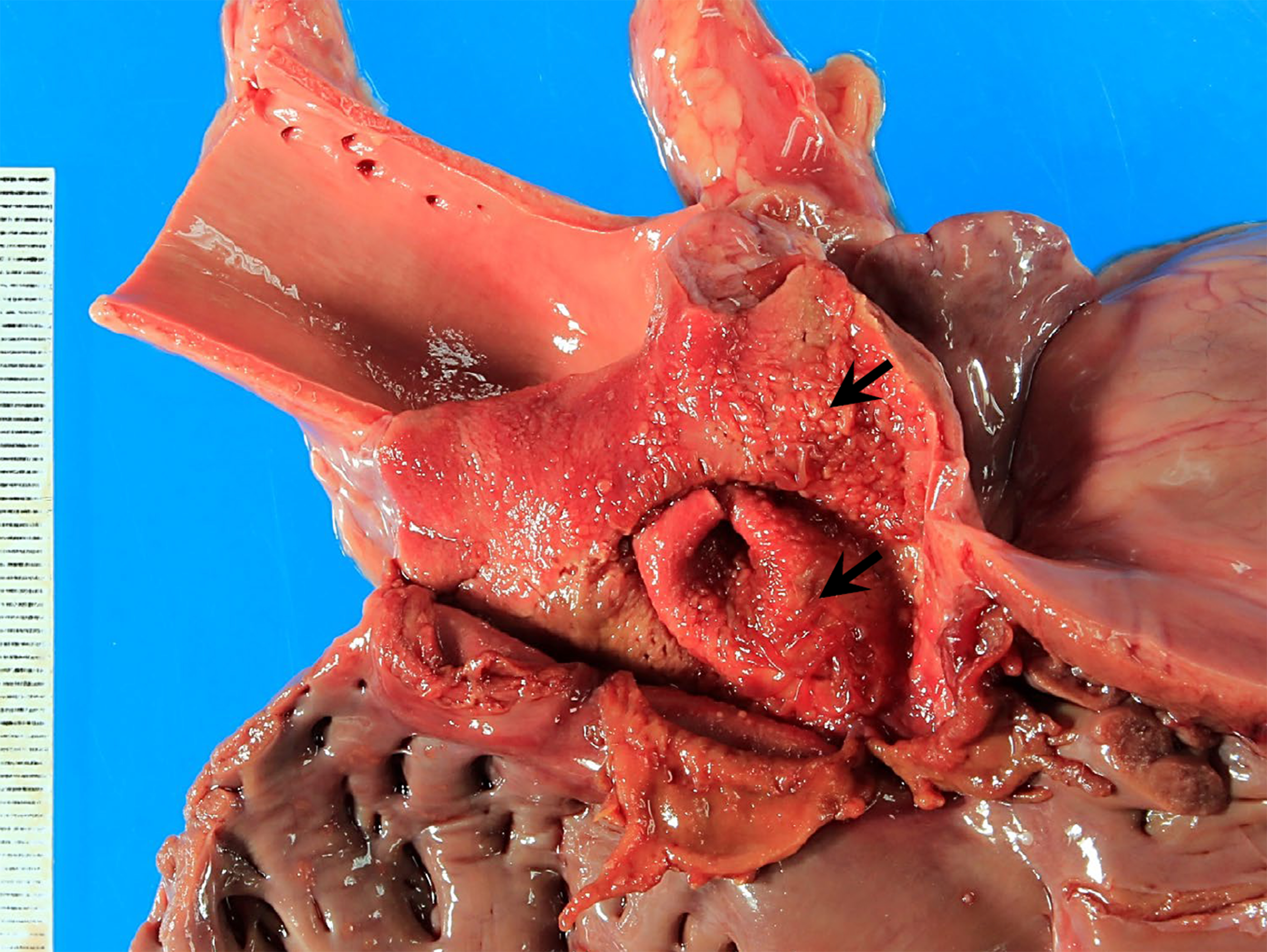

Supplement: Supplementary Figure 3 — The thickening and irregularity of the pulmonic valve and pulmonary trunk were demonstrated (arrow). [file Image_3.jpg]

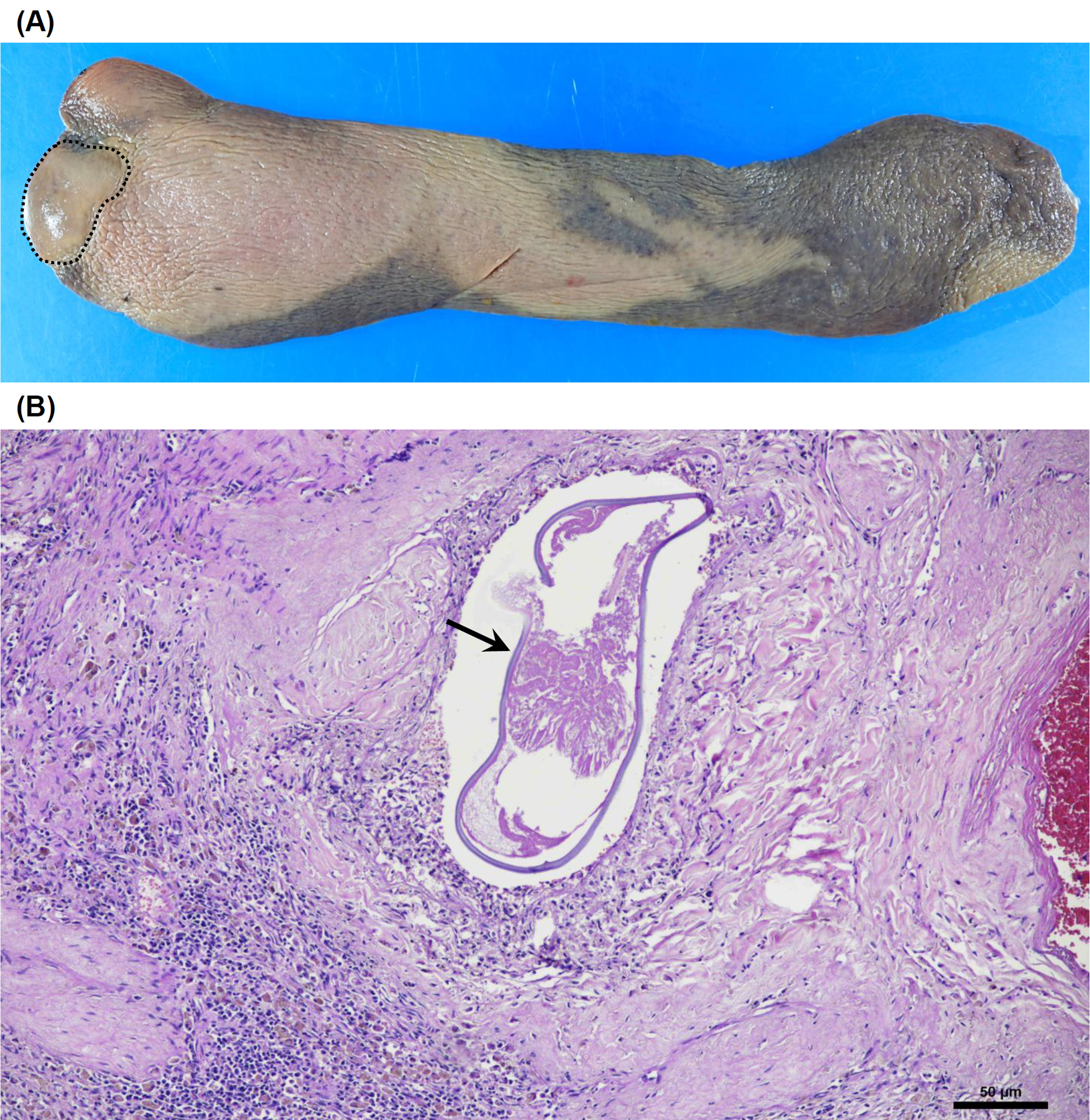

Supplement: Supplementary Figure 4 — Focal infarction (area in the dashed line) was grossly observed at the head of the spleen (A). Microscopically, the occurrence of the cross-sectional adult heartworm parasite in the splenic vessel (arrow). The heartworm was characterized by ~75 μm ×200 in size with 5-μm-thick internal ridges of the cuticle, a well-developed muscle layer, and small guts (B). Bar = 50 μm. [file Image_4.jpg]

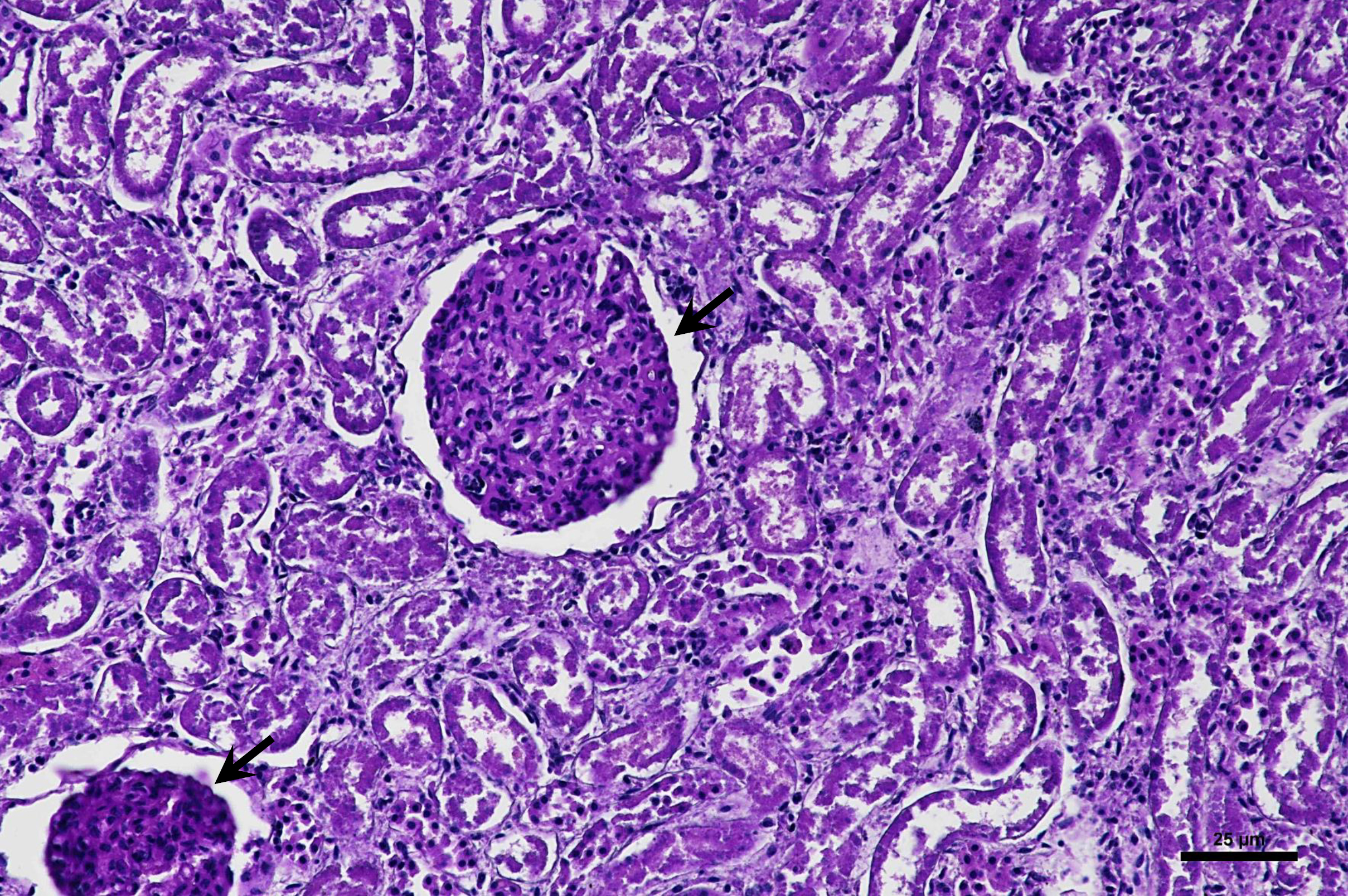

Supplement: Supplementary Figure 5 — Proliferative glomerulonephritis was diffusely observed in the renal cortex of both kidneys. Glomeruli were hyperplasia characterized by increased cellularity and enlarged (arrows). Bar = 25 μm. [file Image_5.jpg]
